# Sparse Evidence of Influenza C on US Dairy and Beef Cattle Farms

**DOI:** 10.1111/irv.70167

**Published:** 2025-10-01

**Authors:** Francisco M. Guerra, Emily M. Edgar, Judith U. Oguzie, Lyudmyla V. Marushchak, Morgan Pattillo, Gregory C. Gray

**Affiliations:** ^1^ Department of Medicine (Division of Infectious Diseases) University of Texas Medical Branch Galveston Texas USA; ^2^ Department of Microbiology and Immunology University of Texas Medical Branch Galveston Texas USA; ^3^ Institute for Human Infections and Immunity University of Texas Medical Branch Galveston Texas USA

**Keywords:** cattle, human, ICV, influenza C, qRT‐PCR, reassortment, whole genome sequencing, zoonotic

Dear Editor,

Recent investigations have demonstrated that influenza C (ICV) is associated with symptomatic bovine respiratory disease (BRD) and that seroprevalence in cattle can be as high as 27% [1]. Prior studies of US cattle in Northwestern and Western states from 2011 to 2014 showed a prevalence of 7.84% [2]. More recently, from areas near North Dakota, the ICV prevalence among cattle decreased to 5.99% [3]. In the present study of Southern US cattle, the ICV prevalence is found to be 0.2%, marking a significant shift. One positive ICV sample from a Texas bovine nasal specimen demonstrated a mixing event between human and bovine ICVs.

Influenza viruses present significant public health and veterinary health concerns due to their genetic variability and potential for widespread transmission through animals and humans [1, 3, 4]. Previously, ICV has primarily affected humans with sporadic spillover to swine and dogs [5, 6, 7]. As humans, we are becoming more connected with farm animals through the food we eat, the people who work with or next to farm animals, and the proximity of farms to cities in the farm‐to‐table movement. These interactions increase the possibility of reservoirs for influenza in multiple species [8]. The One Health concept has demonstrated the interconnected network of health between humans, animals, and the environment [9]. Currently, highly pathogenic avian influenza H5N1, “bird flu,” is raging in wild birds and has jumped species to numerous domestic and wild mammals. As ICV is known to cause infections in humans and pigs, being on the forefront of an outbreak in cattle would well position public health practitioners to prepare for and prevent any potential human pandemics. Investigations of data from Japan from 2020 to 2023 show that ICV had an overall cattle seroprevalence of 27.0% [1]. From 2010 to 2011, 2.32% of children under 10 years of age from 474 mixed human respiratory samples were found to have C/Kanagawa and C/Sao Paulo [10]. In 2009, a novel *influenza A* virus emerged after a reassortment of viruses from different sources including pigs, birds, and humans, which resulted in an influenza pandemic. There is little attention drawn to ICV due to its lower human pathogenicity and prevalence compared to influenza A or B [8]. Consequently, there are no vaccines or effective antiviral treatments currently available for this type of influenza, despite the currently approved polymerase inhibitor baloxavir possessing some antiviral activity against ICV [8, 11]. This study utilizes nasal swab samples collected by the UTMB One Health Team in three US states and one region in Mexico from 2024 to 2025 to estimate the current prevalence of ICV in cattle in North America.

In this study, we conducted qRT‐PCR assays on nasal swabs collected from cattle from dairy farms across three US states (Indiana, Kentucky, and Texas) and one state in Mexico between January 2024 and June 2025 to estimate the current prevalence of ICV in cattle. We used two different primer sets, one that detected the matrix (M) protein and one that detected the nucleoprotein (NP) of ICV. Most of the samples were pooled by farm and field visit. The rest of the samples were tested individually. Approximately 20 cattle nasal samples were collected per farm per visit pending cattle availability. Nasal swab specimens were stored in viral transport medium (VTM), and cold chain was maintained in coolers. In the lab, the samples were quickly transferred to −80°C. RNA extraction was performed on the QIAcube Connect automated extraction system (Qiagen) using the QIAamp Viral RNA Mini Kit (Qiagen, Valencia, CA) following the manufacturer's instructions. qRT‐PCR one‐step assays of the extracted cattle nasal swab samples were performed with one of two primer sets (M or NP genes) using the AgPath‐ID One‐Step RT‐PCR Kit (Cat # AM1005). Extracted RNA pooled sample (5 μL) (or individual sample) was added to a final volume of 25 μL with 0.2‐μM probe and 0.8‐μM forward/reverse primer final concentrations for the M protein assay; remaining volumes were per manufacturer's instructions. Extracted RNA pooled sample (3.5 μL) (or individual sample) was added to a final volume of 21 μL with 0.29‐μM probe and 0.60‐μM forward/reverse primer final concentrations for the NP protein assay; remaining volumes were per manufacturer's instructions. Cycling conditions for the M protein assay were 50°C for 10 min, 95°C for 10 min, and 45 cycles of 95°C for 45 s with 60°C for 15 s. The NP assay was similar with 48°C for 10 min, 95°C for 10 min, and 45 cycles of 95°C for 15 s with 60°C for 45 s. The single positive sample detected from the qRT‐PCR assays was isolated and grown in MDCK cells. RNA from the single laboratory reproduced positive sample was extracted and prepared for next generation sequencing (NGS).

Sequencing libraries were made using the NEB Next Ultra II RNA Library Prep Kit for Illumina (New England Biolabs, Ipswich, MA) in accordance with the manufacturer's instructions for the sample that was positive for ICV. A 75‐bp paired‐end NGS was carried out on Element Biosciences' AVITI Sequencer platform available at https://www.elementbiosciences.com/. Raw sequencing reads were analyzed on the Chan Zuckerberg ID metagenomic platform (CZ ID platform) available at https://czid.org/. A consensus genome for the single positive ICV sample was assembled after taxon classification and host refinement. Phylogenetic analysis was conducted using 1000 HEF genes.

From surveilling 433 cattle from farms across three US states and one state in Mexico from 2024 to 2025, using qRT‐PCR assays, we detected one positive case of bovine ICV representing a 0.2% prevalence. NGS of the positive ICV bovine nasal swab sample showed that Segments 1–6 had 96%–98% nucleotide sequence identity with C/Bovine/Montana/12/2016. Segment 7, on the other hand, which encodes NS1 and NS2, showed ~98% nucleotide sequence identity with C/Mississippi/80, which is a human‐derived ICV variant (Table 1). Based on the hemagglutinin‐esterase‐fusion (HEF) gene sequence on Segment 4, our genome is overall most closely related to C/Bovine/Montana/12/2016 (Table 1).

**TABLE 1:** Bovine nasal swab positive sample whole genome analysis.

Sequencing ID	Percent identity	Best BLAST hit (accession number)	Best BLAST hit (country)	Best BLAST hit (host)	Hit annotation on NCBI
USL097_TX_seg1_PB2	97.64	MH348113.1	Montana, USA	Bovine	Influenza C virus (C/Bovine/Montana/12/2016) Segment 1 polymerase PB2 (PB2) gene, complete cds
USL097_TX_seg2_PB1	98.35	MH348114.1	Montana, USA	Bovine	Influenza C virus (C/Bovine/Montana/12/2016) Segment 2 polymerase PB1 (PB1) gene, complete cds
USL097_TX_seg3_P3	98.0	MH348115.1	Montana, USA	Bovine	Influenza C virus (C/Bovine/Montana/12/2016) Segment 3 polymerase P3 (P3) gene, complete cds
USL097_TX_seg4_HE	98.83	MH348116.1	Montana, USA	Bovine	Influenza C virus (C/Bovine/Montana/12/2016) Segment 4 hemagglutinin‐esterase (HE) gene, partial cds
USL097_TX_seg5_NP	96.74	MH348117.1	Montana, USA	Bovine	Influenza C virus (C/Bovine/Montana/12/2016) Segment 5 nucleoprotein (NP) gene, complete cds
USL097_TX_seg6_M1 and CM2	97.08	MH348118.2	Montana, USA	Bovine	Influenza C virus (C/Bovine/Montana/12/2016) Segment 6 Matrix Protein 1 (M1) and CM2 protein (CM2) genes, complete cds
USL097_TX_seg7_NS1 and NS2	97.82	LC124982.1	Mississippi, USA	*Homo sapiens*	Influenza C virus (C/Mississippi/80) NS1, NS2 genes for Nonstructural Protein 1, Nonstructural Protein 2, complete cds

ICV contains seven segments encoding nine proteins [5]. ICV has one surface glycoprotein, HEF, which combines hemagglutinin and neuraminidase functions onto a single fusion protein [1]. ICV binds to receptors displaying 9‐*O*‐acetyl‐*N*‐acetylneuraminic acid‐bearing receptors [6]. Sialic acid α‐2,3‐ and α‐2,6‐galactose host receptors, the receptor targets of avian and human influenza A, have been found in cattle mammary glands [12]. The surveillance in this study found 0.2% among 433 sample cattle nasal swabs. In comparison, after homogenization and extraction of viral RNA from 217 pneumonia positive lung samples from cattle necropsies submitted to South Dakota State University from 2019 to 2020, ICV prevalence was found to be 5.99% [3]. In another study, including farms from NM, CO, and WA states from 2011 to 2014, 7.84% overall prevalence among 599 was detected [2]. This present study, with 0.2% ICV prevalence, represents a 10‐fold decrease in the prevalence of ICV. Cases of influenza D (IDV) in concurrent studies (published separately by the UTMB One Health Gray laboratory) show a higher prevalence of IDV in comparison to ICV. There is a geographic difference between the farms sampled in this study (Mexico, TX, KY, and IN) versus the Southwestern and Northwestern US states in the other studies. The other studies are also 5–14 years old, which could mean there has been ample time for ICV numbers to dwindle.

NGS analysis of the positive C/Bovine/Texas/2024 bovine nasal swab sample showed that Segment 7 (encoding NS1 and NS2) showed 98% sequence identity with C/Mississippi/80. Nonstructural Protein 1 (NS1) is a ~230‐amino acid interferon antagonist with multiple functions [6]. Nonstructural Protein 2 (NS2) is a 121‐amino acid RNP nuclear export protein (NEP) that helps regulate RNA synthesis [6]. NS1 is known to inhibit Type 1 interferon gamma (IFN) production, which dampens the host innate immune response to respiratory viral infections [13, 14]. Sequence analysis of C/Bovine/Texas/2024 shows overall high sequence similarity with C/Bovine/Montana/12/2016.

Analysis of the single positive ICV sample suggests there has been genetic reassortment between human versions and bovine versions of ICV. Due to limited data available for ICV, there is still a chance that the virus we identified is novel bovine ICV that has been circulating in cattle. As has been observed with influenza A, genetic drift in the ICV HEF protein could render the virus better adapted to human hosts and ultimately more pathogenic. The longer ICV stably infects industrial cattle herds, the more time the virus has to adapt to mammalian hosts; this is true for all cattle viruses but particularly influenza A and C. The relative novelty of ICV poses a barrier to analyzing the progression of this virus through a population due to lack of data. Surveillance of ICV is crucial to ensure that potential reservoirs are monitored for potential outbreaks or genetic differences in the host population [8]. ICV possesses the capability to generate illnesses that pose an issue to human and animal health. This study highlights the importance of sampling and testing agricultural areas to record the activity of ICV.

## Author Contributions

**Francisco M. Guerra Jr:** investigation, writing – original draft, methodology, validation, writing – review and editing, formal analysis, data curation, supervision. **Emily M. Edgar:** investigation, methodology, formal analysis, data curation, writing – original draft, writing – review and editing. **Judith U. Oguzie:** investigation, writing – original draft, writing – review and editing, methodology, formal analysis, software, data curation. **Lyudmyla V. Marushchak:** investigation, methodology, validation, formal analysis, supervision. **Morgan Pattillo:** investigation, methodology. **Gregory C. Gray:** conceptualization, investigation, funding acquisition, writing – review and editing, methodology, formal analysis, supervision, resources, project administration.

## Conflicts of Interest

The authors declare no conflicts of interest.

## Data Availability

The data that support the findings of this study are available from the corresponding author upon reasonable request.
